# The Associations Between Gallstone Disease and Pan‐Cancer Incidence Risk Based on Over 13 Million Participants

**DOI:** 10.1002/cam4.70857

**Published:** 2025-04-25

**Authors:** Wenqian Yu, Jin Zhou, Jing Luo, Jing Xia, Shiyi Li, Linjun Xie, YaZhou He, Hongyu Li, Guoheng Jiang, Xin Chen, Xuan Bai, Min Mao, Xin Wang

**Affiliations:** ^1^ Department of Epidemiology and Biostatistics, West China School of Public Health and West China Fourth Hospital Sichuan University Chengdu P. R. China; ^2^ West China‐PUMC C. C. Chen Institute of Health, West China School of Public Health and West China Fourth Hospital Sichuan University Chengdu P. R. China; ^3^ Department of Anorectal Surgery Hospital of Chengdu University of Traditional Chinese Medicine Chengdu Sichuan Province P. R. China; ^4^ Department of Oncology, West China School of Public Health and West China Fourth Hospital Sichuan University Chengdu P. R. China; ^5^ Department of Pediatric Pulmonology and Immunology, West China Second University Hospital Sichuan University Chengdu P. R. China; ^6^ Key Laboratory of Birth Defects and Related Diseases of Women and Children (Sichuan University), Ministry of Education Chengdu P. R. China; ^7^ NHC Key Laboratory of Chronobiology (Sichuan University) Chengdu P. R. China; ^8^ The Joint Laboratory for Lung Development and Related Diseases of West China Second University Hospital, Sichuan University and School of Life Sciences of Fudan University, West China Institute of Women and Children's Health, West China Second University Hospital Sichuan University Chengdu P. R. China; ^9^ Sichuan Birth Defects Clinical Research Center, West China Second University Hospital Sichuan University Chengdu P. R. China

**Keywords:** all‐cause of cancer risk, gallstone disease, meta‐analysis, risk

## Abstract

**Background:**

Increasing evidence connects gallstone disease (GSD) to all types of cancer incidence; however, the results were inconsistent. The present study aimed to evaluate whether and to what extent these associations exist comprehensively.

**Methods:**

We systematically searched published longitudinal studies indexed in PubMed and Embase database from dates of inception to March 31, 2020. We pooled the effect of GSD on all‐cause cancer incidence. Moreover, we further employed stratified analysis concerning sex, geographic background, surgery status, and follow‐up period. Trial sequential analysis (TSA) was applied to decide whether the included sample size was sufficient for evaluating these associations.

**Results:**

Fifty‐one studies incorporating over 13 million participants were eligible for analysis in this study. GSD pose an increased risk of all‐cause cancer risk (pooled RR = 1.43; 95% CI: 1.33–1.54) compared with the healthy controls, especially hematologic malignancy (pooled RR = 1.14; 95% CI: 1.05–1.25), gastrointestinal cancers (pooled RR = 1.28; 95% CI: 1.15–1.41), liver, pancreas, and biliary tract cancer (pooled RR = 1.84; 95% CI: 1.62–2.10), and kidney cancer (pooled RR = 1.19; 95% CI: 1.03–1.37). These associations are not markedly changed after stratification by different subgroups. Moreover, the TSA confirmed the sample size was sufficient to draw these conclusive conclusions.

**Conclusions:**

The present meta‐analysis with sufficient evidence indicates GSD increases the risk for all causes of cancer incidence. The evidence may warrant GSD patients to perform screening and prophylactic treatment for the prevention of these complications. The indication for cholecystectomy should be determined through a comprehensive evaluation of the patient's clinical presentation, with a thorough assessment of the potential therapeutic benefits and surgical risks.

## 1 Introduction

Gallstone disease (GSD) is one of the widespread gastrointestinal conditions and a leading inpatient admission cause across the globe [1]. Overall, up to 20% of adults were reported to harbor GSD [2]. Moreover, with the ongoing pandemic rise of metabolic abnormalities, as well as unhealthy life and diet patterns, the prevalence of GSD was expected to increase in the foreseeable future persistently [2]. Throughout the natural course of GSD, approximately 20% of patients develop biliary manifestations or related complications necessitating surgical intervention. This clinical observation suggests that the majority of affected individuals remain asymptomatic and undiagnosed, with a potential risk of disease progression to symptomatic GSD in subsequent years [2]. It is reported that over 50,000 cholecystectomies are performed each year in the United Kingdom. While in the United States, cholecystectomies approximately reach 800,000 and consume nearly 6.0 billion dollars annually, which inflicts a heavy health burden and economic costs [3, 4, 5].

Accumulating epidemiological evidence suggests that the pathological implications of GSD extend beyond the hepatobiliary system, with multiple cohort studies demonstrating significant associations between GSD and increased risk of various malignancies, including leukemia [6], gastric cancer [7, 8], colorectal cancer [9, 10], pancreatic cancer [11], and gallbladder cancer [12, 13]. However, due to the differences in the sample size, population stratification, and the length of follow‐up, conflicting results also exist [14, 15, 16].

Having a better look into the associations between cancer incidence and GSD may provide clues for clinicians to tailor treatment regimens and screening schemes for the co‐occurrence of cancer. However, owing to the scarcity of consistent and robust evidence, it is impossible to provide any advisable strategies. In this scenario, we aim to comprehensively evaluate the effect of GSD on all‐cause cancer incidence. Moreover, we also try to estimate whether these associations differ between sexes, diverse ethnic backgrounds, surgery status (with or without cholecystectomy), and different follow‐up periods through systematic review and meta‐analysis. Additionally, we also designed to appraise whether the current existing pieces of evidence are sufficient and conclusive using trial sequential analysis (TSA) [17].

## 2 Methods

### 2.1 Literature Search

We systematically searched published literature indexed in PubMed and Embase from their dates of inception to March 31, 2020. The current systematic review and meta‐analyses have been registered with the International Prospective Register of Systematic Reviews (PROSPERO registration number: CRD42020160360). The information for the literature search, study selection, and data extraction was listed in Appendix S1.

### 2.2 Study Selection

Two authors independently evaluated the identified titles and abstracts of all citations. The inclusion criteria were listed as follows: (i) longitudinal study design including cohort study, nested case–control research, case‐cohort study, and follow‐up study of randomized controlled trials; (ii) they evaluated the associations between GSD and incidence of all cancers; (iii) articles provided hazard ratio (HR), odds ratio (OR), risk ratio (RR), standardized incidence ratio (SIR), or standard morbidity ratio (SMR) and its 95% confidence interval (CI) or provided sufficient data for calculating the effect size with 95% CI; (iv) GSD was defined as the presence of gallstones or history of cholecystectomy; (v) the participants without GSD or the GSD patients without cholecystectomy were used as the reference group; (vi) when multiple publications included the same population, studies with larger sample size or more extended follow‐up period were incorporated. References of the retrieved articles were also manually checked to identify additional studies that could not be captured by the initial literature search strategy.

### 2.3 Data Abstraction

Two reviewers independently extracted the data, and it was subsequently double‐checked by two others. Data extracted from each eligible publication concerning the first author, publication year, geographic background, follow‐up period, study population, surgery status (with or without cholecystectomy), sample size, mean age, study period, effect size (OR, HR, RR, SIR or SMR) and 95% CI from the multivariable model with confounding factors adjusted. Any disagreements were resolved by consensus.

### 2.4 Statistical Analyses

Due to the relatively low incidence of the events mentioned above, the effect sizes of OR, HR, RR, SIR, and SMR were deemed identical in this meta‐analysis [18]. When calculating the effect size for studies with zero events in the 2 × 2 table, the continuity correction method conducted by Sweeting and colleagues was applied [19]. Cochran's *Q*‐test evaluated across study heterogeneity. *I* ^2^ assessed the amount of variance due to heterogeneity. If heterogeneity was evident (*p* for *Q*‐test < 0.05), the DerSimonian and Laird random‐effects model was performed, while the fixed effects model was used if *p* was higher than 0.05 [20]. Stratified analysis was further employed to investigate the associations between GSD and outcomes in the subgroups concerning sex and geographic background. We also conducted a subgroup analysis to investigate the associations stratified by surgery status (with vs. without cholecystectomy). Moreover, on account of the time‐lag bias and to trace the time‐course effect of GSD on the outcomes, we performed another subgroup analysis stratified by different follow‐up year cutoffs [21]. Mixed‐effects meta‐regression was further conducted to test the trend of the effect size changes with the follow‐up year. We additionally conducted a sensitivity analysis by iteratively omitting each dataset at a time to examine the stability of the overall pooled results. Publication bias was evaluated by visual inspection of the asymmetry of funnel plots, Egger's, and Begg's tests approaches [22, 23]. The Duval and Tweedie trim‐and‐fill method also adjusted pooled effects by generating hypothetical missing studies [24].

Due to the sparse data and repetitive tests of accumulative studies, meta‐analyses may result in Type I error and provide a false‐positive finding [25]. Therefore, TSA was applied to decide whether the sample size incorporated into the meta‐analysis was sufficient for evaluating the associations [17]. Significant associations exist when the Z‐curve crosses the conventional boundary. If the Z‐curve passes through the TSA monitoring boundary or required information size (RIS) boundary, the evidence is considered sufficient and conclusive. Otherwise, the association is inconclusive, and more studies are needed to verify the results further.

We synthesized the pooled effect size and 95% CIs using Comprehensive Meta‐Analysis version 2.0 (Biostat, Englewood, NJ, USA). A summarized forest plot was drawn using R version 3.4.1 (R Foundation for Statistical Computing, Vienna, Austria; www.r‐project.org). The TSA was conducted by TSA program version 0.9 beta (Copenhagen Trial Unit, Centre for Clinical Intervention Research, Copenhagen, Denmark, 2011).

## 3 Results

### 3.1 Literature Selection

**FIGURE 1:**
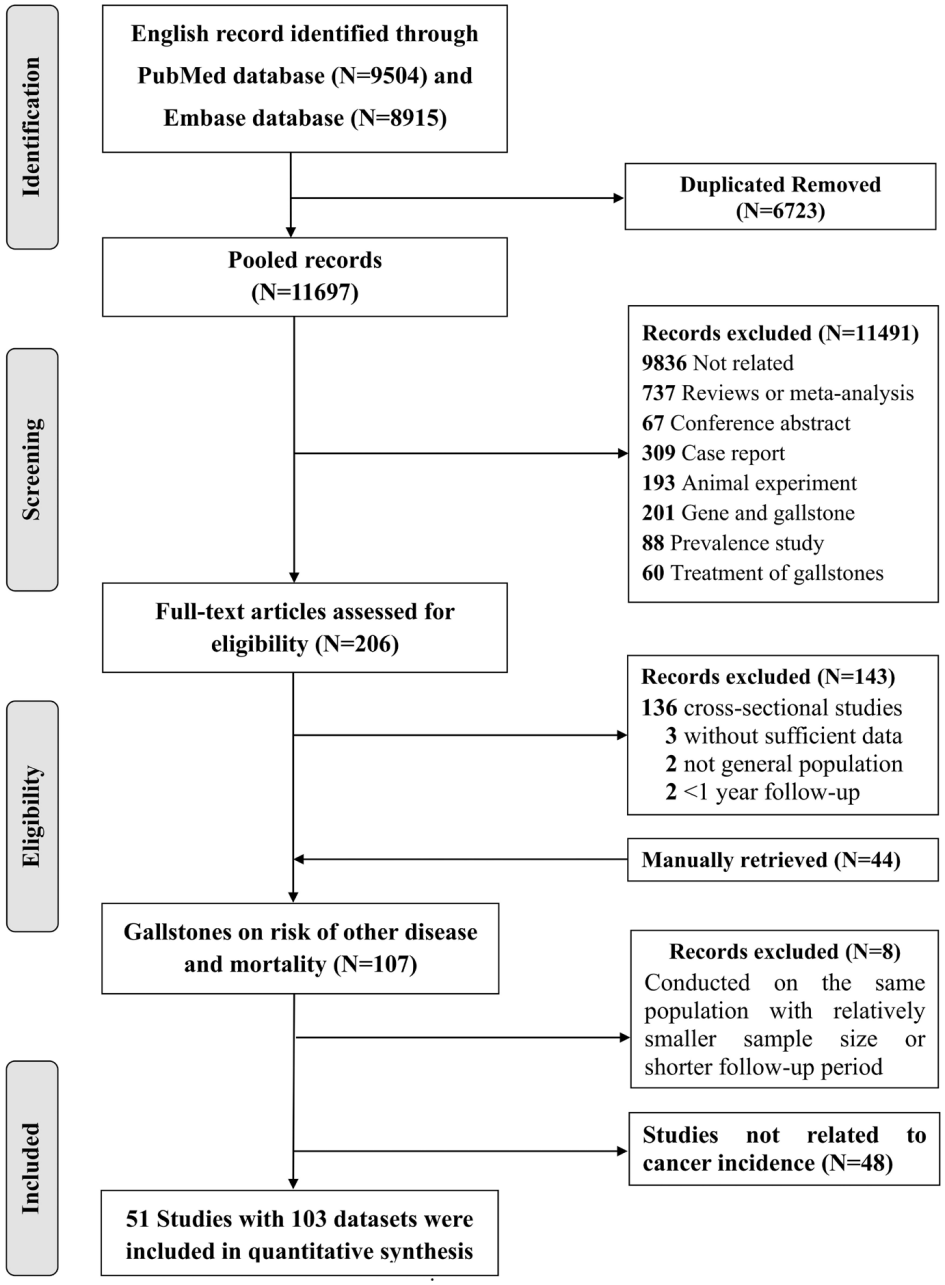
Flowchart of the study selection procedure of the present study. After excluding duplicates, studies with irrelevant content, reviews and meta‐analyses, studies with missing data, and studies with smaller sample sizes or shorter follow‐up periods within the same cohort, a total of 51 articles were ultimately included in this study.

The flowchart of the study screening and selection process is presented in Figure 1. Overall, 18,419 records were initially identified through the PubMed and Embase databases. After carefully reviewing titles and abstracts, we included a total of 107 studies to investigate the effect of GSD on cancer incidence. However, eight studies were excluded due to a relatively minor sample or shorter follow‐up period compared with other included studies. Moreover, 48 studies were further excluded because they were not focusing on the associations between GSD and cancer incidence. Finally, 51 cohort studies (with 103 datasets) incorporating approximately 14 million participants (*N* = 13,884,465) were eligible for analysis in the present study (Appendix S2).

### 3.2 Study Characteristics

Of the included 51studies, four reported the effect of GSD on eye and nervous system cancer (Appendix S2; A1, A8, A11, A20), two on head and neck cancer (A8, A11), four on hematologic malignancy including lymphoma and myeloma cancer and leukemia (A1, A8, A11, A20), three on lip, oral cavity, and pharynx cancer (A1, A9, A28), four on skin cancer (A1, A8, A11, A20), 19 on gastrointestinal cancer including esophageal cancer, gastric cancer, small bowel cancer, and colorectal cancer (A1–A4, A8, A9, A11, A14, A15, A17, A20–A25, A27, A31, A35), 22 on liver, biliary and pancreatic cancer (A5–A11, A12, A13, A16, A18–A20, A26, A29, A32, A33, A36, A39, A44, A45, A47), four on urinary cancer including kidney cancer and bladder cancer (A1, A8, A11, A20), five on respiratory cancer including lung cancer and laryngeal cancer (A1, A8, A11, A20, A28), one on bone, connective, and muscle cancer (A11), six on male specific cancer including prostate cancer, testis and penis cancer (A1, A5, A8, A11, A20, A34), and six on female specific cancer including breast cancer, cervical cancer, ovary cancer, uterine corpus cancer, vagina, and vulva cancer (A1, A5, A8, A11, A20, A30). Moreover, five studies study the effect of cholecystectomy on cancer incidence (A6, A8, A48–A50) compared with GSD patients without cholecystectomy (Table 1). Detailed characteristics of the included studies are displayed in Appendix S3.

**TABLE 1:** Baseline characteristics for studies included in meta‐analysis.

Type of groups	No. of included datasets	Geographic background			Sample size	Mean Follow‐up (years)
		America	Europe	Asia		
Gallstone disease vs subjects without gallstones						
Cancer incidence	48	10	27	11	13,309,278	3.3~30.0
Eye and nervous system cancer	4	1	3	0	494,358	7.4~30.0
Lip, oral cavity, and pharynx cancer	3	0	3	0	387,817	7.4~30.0
Head and neck cancer	2	0	1	1	78,193	11.0~30.0
Hematologic malignancy	4	0	3	1	494,358	7.4~30.0
Skin cancer	4	0	3	1	494,358	7.4~30.0
Gastrointestinal cancer	29	8	17	4	4,417,670	3.3~30.0
Liver, pancreas and biliary tract cancer	22	4	11	7	9,180,506	5.0~30.0
Urinary cancer	4	0	3	1	494,358	7.4~30.0
Respiratory cancer	5	0	4	1	839,609	7.4~30.0
Bone, connective, and muscle cancer	1	0	1	0	468	30.0
Male specific cancer	6	0	4	2	258,623	7.4~30.0
Female specific cancer	6	0	5	1	546,783	3.8~30.0
Cholecystectomy vs. GSD patients without surgery						
Cancer incidence	5	0	0	5	1,201,399	2.4~11.0
Gastrointestinal cancer	3	0	0	3	575,499	2.4~11.0
Liver, pancreas, and biliary tract cancer	2	0	0	2	496,233	11.0
Prostate cancer	1	0	0	1	145,212	5.5–5.7

### 3.3 Main Meta‐Analysis

**FIGURE 2:**
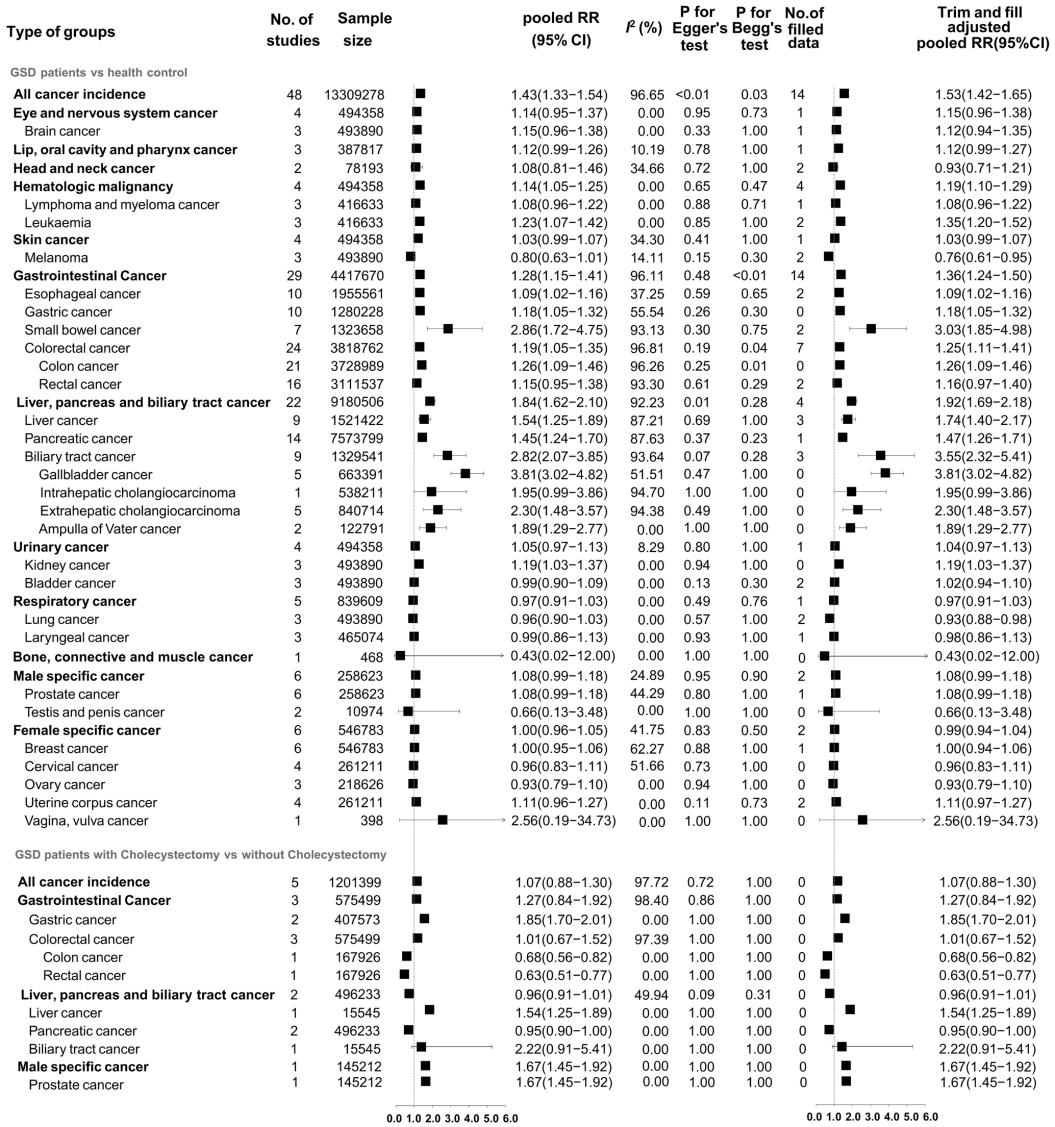
The forest plot for the associations between gallstone disease and all‐cause of cancer incidence and the publication bias test with trim and fill adjusted results. Type of groups: the types of cancer; No. of studies: the number of studies included; Sample size: the sample size encompassed by these studies; Pooled RR (95% CI): the RR and its 95% CI pooled based on the original data; *I* ^2^: Quantify the heterogeneity between studies, where a higher value indicates greater heterogeneity among the studies. *P* for Egger's test or Begg's test < 0.05 indicates that there may be publication bias; No. of filled data: the number of filled studies based on the Duval and Tweedie trim‐and‐fill method; Trim and fill adjusted pooled RR (95% CI): the pooled RR and its 95% CI adjusted by the Duval and Tweedie trim‐and‐fill method.

When compared with the health subjects, the present meta‐analysis suggested GSD patients have an increased risk of all‐cause cancer occurrence (pooled RR = 1.43; 95% CI: 1.33–1.54) (Figure 2). For detailed types of cancer, results indicated that GSD poses a higher risk of hematologic malignancy (pooled RR = 1.14; 95% CI: 1.05–1.25, mainly leukemia: pooled RR = 1.23; 95% CI: 1.07–1.42), gastrointestinal cancers (pooled RR = 1.28; 95% CI: 1.15–1.41, especially the esophageal cancer: pooled RR = 1.09; 95% CI: 1.02–1.16; the gastric cancer: pooled RR = 1.18; 95% CI: 1.05–1.32; small bowel cancer: pooled RR = 2.86; 95% CI: 1.72–4.75; colon cancer: pooled RR = 1.26; 95% CI: 1.09–1.46), liver, pancreas, and biliary tract cancer (pooled RR = 1.84; 95% CI: 1.62–2.10; mainly the liver cancer: pooled RR = 1.54; 95% CI: 1.25–1.89; pancreatic cancer: pooled RR = 1.45; 95% CI: 1.24–1.70; gallbladder cancer: pooled RR = 3.81; 95% CI: 3.02–4.82; extrahepatic cholangiocarcinoma: pooled RR = 2.30; 95% CI: 1.48–3.57; ampulla of cater cancer: pooled RR = 1.89; 95% CI: 1.29–2.77), and kidney cancer (pooled RR = 1.19; 95% CI: 1.03–1.37). We found significant publication bias in all‐cancer incidence. The trim and fill method showed significant associations between GSD and melanoma (adjusted pooled RR = 0.76; 95% CI: 0.61–0.95) and lung cancer (adjusted pooled RR = 0.93; 95% CI: 0.88–0.98), while pooled effect sizes for other disease outcomes did not significantly change after adjustment. However, the data are insufficient to draw significant associations between GSD and other cancer types (Figure 2).

For the GSD patients, results showed the cholecystectomy could not significantly reduced the all‐cause cancer occurrence risk compared with the GSD patients without surgery (pooled RR = 1.07; 95% CI: 0.88–1.30). With respective to different cancer types, results suggested the cholecystectomy could significantly reduce the risk of colon cancer (pooled RR = 0.68; 95% CI: 0.56–0.82), rectal cancer (pooled RR = 0.63; 95% CI: 0.51–0.77), and pancreatic cancer occurrence (pooled RR = 0.95; 95% CI: 0.90–1.00), while the cholecystectomy could significantly increase the gastric cancer (pooled RR = 1.85; 95% CI: 1.70–2.01), liver cancer (pooled RR = 1.54; 95% CI: 1.25–1.89), and prostate cancer risk (pooled RR = 1.67; 95% CI: 1.45–1.92). However, the cholecystectomy was not significantly associated with the biliary tract cancer risk (pooled RR = 2.22; 95% CI: 0.91–5.41) (Figure 2).

### 3.4 Subgroup and Meta‐Regression Analysis

**FIGURE 3:**
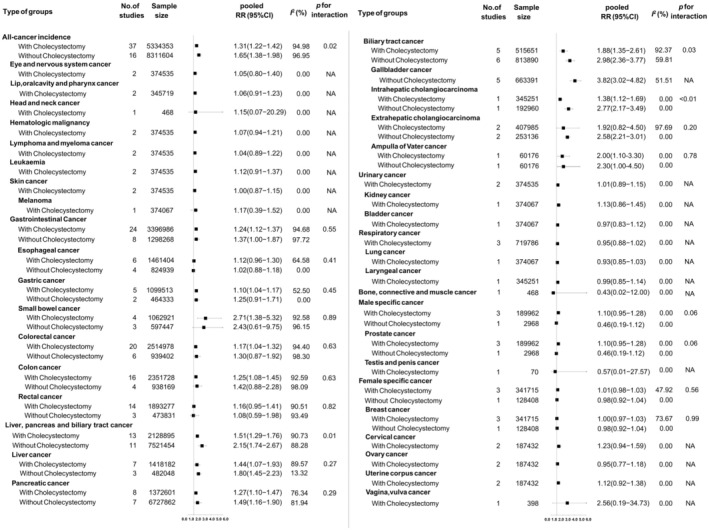
The forest plot for the associations between surgery status of gallstone disease and all‐cause of cancer incidence. Type of groups: The types of cancer; No. of studies: the number of studies included; Sample size: the sample size encompassed by these studies; Pooled RR (95% CI): the RR and its 95% CI pooled based on the original data; *I* ^2^: Quantify the heterogeneity between studies, where a higher value indicates greater heterogeneity among the studies. *p* for interaction: Assess whether the differences in effect sizes between different subgroups are statistically significant, *p* for interaction < 0.05 means that the differences between subgroups are considered statistically significant.

**FIGURE 4:**
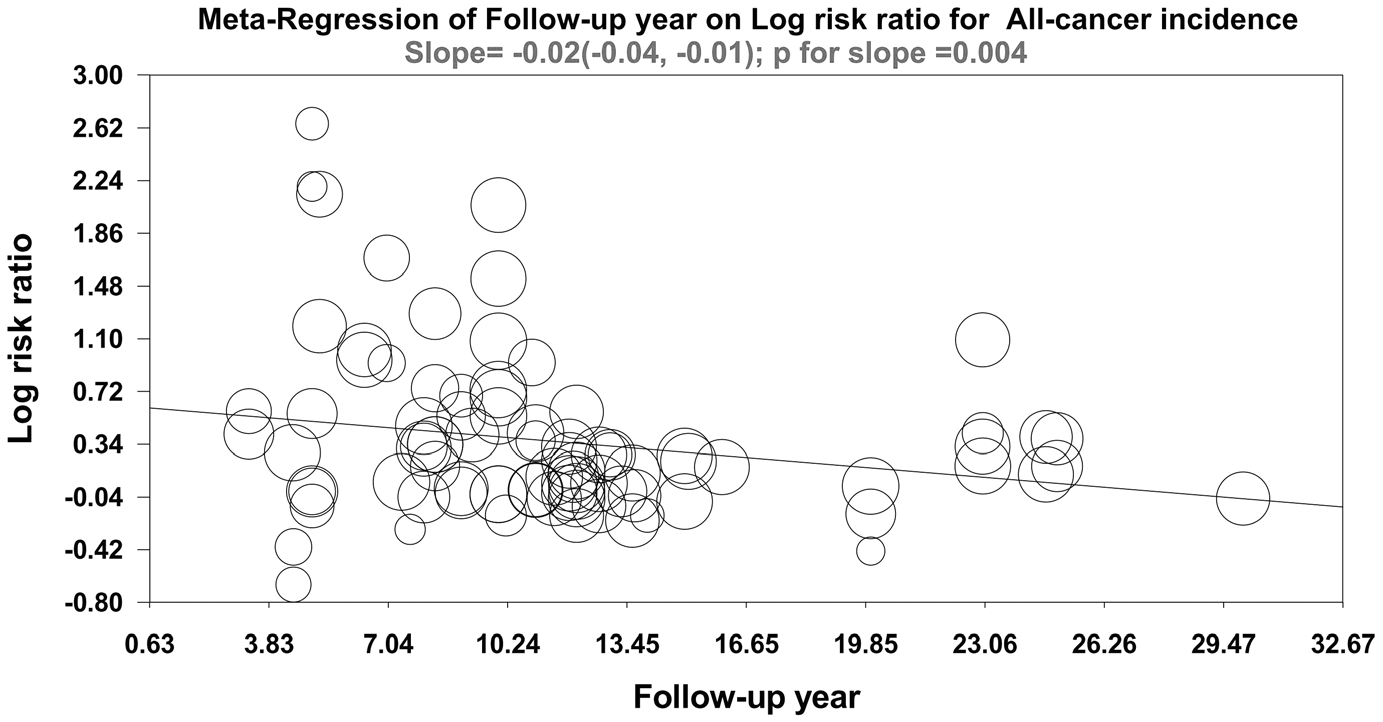
Meta‐regression plot of follow‐up years on the log risk ratio of all‐cancer incidence. The negative slope indicates an inverse correlation between follow‐up year and log risk ratio. And *p*‐value for slope < 0.05 suggests that the influence of follow‐up year on log risk ratio is significant.

Sensitivity analysis showed that no study substantially affected the results (Appendix S4). When stratified by surgery status (Figure 3), gender (Appendix S5) and geographic background (Appendix S6), the heterogeneity was all decreased to some extent, and the associations were not significantly changed. Meta‐regression analysis suggested with the follow‐up period increased, the strength of associations between GSD and all cause of cancer risk was significant decreased (Figure 4). Additionally, when stratified by different follow‐up year periods, these associations were also significant. The magnitude of the associations in each follow‐up duration is similar, indicating no significant influence of reverse causality and lag‐time bias (Appendix S7).

### 3.5 TSA For Evaluating the Reliability of the Evidence

**FIGURE 5:**
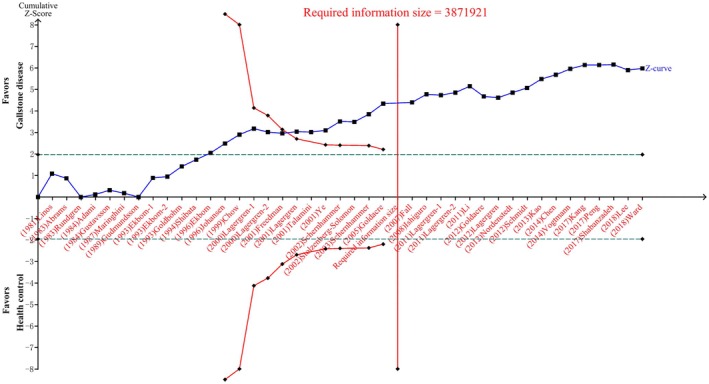
Trial sequential analysis for estimating the reliability of evidences available for the associations between gallstone disease and all cause of cancer risk. The line that shows an upward trend as the number of studies increases represents the Z‐curve. The horizontal line parallel to the x‐axis is the conventional significance boundary. The TSA monitoring boundary gradually converges toward the conventional significance boundary as the cumulative sample size or number of events increases. The vertical line intersecting the x‐axis represents required information size and indicates the minimum sample size or number of events required to achieve sufficient statistical power.

In the TSA, the cumulative Z curve passed through the futility boundary and achieved RIS boundary for all‐cancer incidence, which indicated the associations between GSD and all cause of cancer occurrence risk was sufficient and conclusive (Figure 5).

For different cancer types, the Z score both crossed the conventional boundary and RIS boundary for hematologic malignancy (Appendix S8D), gastrointestinal cancer (Appendix S8F) and liver, biliary, and pancreatic cancer (Appendix S8G), which indicates that the available evidence is sufficient to suggest positive associations. Moreover, the Z score crossed the RIS boundary before reaching the conventional boundary for the associations between GSD and male and female specific cancer risk, indicating the relatively strong evidence of no associations between them (Appendix S8J–K). However, the cumulative z‐curve did not reach the conventional test boundary and RIS for eye and nervous system cancer, lip, oral cavity, and pharynx cancer, headache and neck cancer, skin cancer, and urinary cancer, making these results inconclusive but encouraging for more studies to validate these associations (Appendix S8A–C,E,H).

## 4 Discussion

### 4.1 Summary

The current meta‐analysis incorporated over 13 million participants to evaluate the effect of GSD on all types of cancer occurrence risk. Collectively, results demonstrate that a history of GSD gives an increased risk of all cancer incidences, especially hematologic malignancies, gastrointestinal cancer, liver, biliary and pancreatic cancer, and kidney cancer. These associations did not markedly change after being stratified by sex, geographic background, and surgery status. Moreover, the TSA also confirmed the sufficiency and conclusiveness of the associations. Additionally, compared with GSD patients without surgery, cholecystectomy gives risk to gastric cancer, liver cancer, and prostate cancer.

Recent advancements in epidemiological research have yielded multiple meta‐analyses examining the association between GSD and oncological risk. Notably, Huang et al., Li et al., and Polychronidis et al. have systematically evaluated the correlation between GSD and the incidence of biliary tract cancer, prostate cancer, and colorectal cancer through comprehensive analyses of existing cohort and case–control studies [26, 27, 28]. These findings have been further substantiated by a 2021 Mendelian randomization study [29], demonstrating a significant causal relationship between GSD and elevated risk of these cancers. Our results are consistent with these established findings, confirming GSD as a significant risk factor for these cancer types. However, the study by Sun et al. revealed a distinct geographical variation, with a significant association between GSD and pancreatic cancer risk observed exclusively in the Asian population subgroup analysis [30]. This observation aligns with our findings, which demonstrate a more pronounced effect of GSD on pancreatic cancer risk in the Asian subgroup (*p* for interaction < 0.01). While recent meta‐analyses have provided valuable updates on specific cancer types, our study offers a more comprehensive evaluation of heterogeneity sources, incorporating critical variables such as sex, geographical background, and surgical intervention status. The results demonstrate remarkable consistency with existing literature while providing additional insights into potential effect modifiers.

### 4.2 Mechanisms

This study found a positive association between GSD and hematologic malignancy, especially leukemia. But due to the relatively smaller sample size, the association was different when stratified by sex and country subgroups, and the association just persisted during 5.1~10.0 years of follow‐up subgroup; it could not be validated by longer follow‐up. Thus, the association may be caused by an underlying undiagnosed disease in the baseline investigation, and more studies are needed to validate the association further [21].

Results support positive associations between GSD and gastrointestinal cancer, especially the gastric, small bowel, and colon cancer risk. It may be partly explained by the coexistence of risk factors for cancer and GSD, such as obesity and alcohol consumption [31]. Moreover, results also showed that subjects with cholecystectomy suggested having relatively higher credible evidence for developing gastrointestinal cancer. For the possible reason that the gallbladder is removed, the mechanism may assist the association; biliary fluids may continuously flow into the duodenum and increase the duodenogastric bile reflux [32]. Moreover, continuous exposure to bile may lead to increased bacterial degradation, which accelerates the production of more secondary bile acids and promotes carcinogenesis [33]. However, the association between cholecystectomy and colorectal adenomas, regarded as the precursor to colorectal cancer, was not significant [34]. The reason may be explained by the relatively smaller sample size, and more studies with larger samples are needed to confirm the association. Additionally, results showed that when compared with GSD patients without surgery, cholecystectomy was not associated with gastrointestinal cancer risk anymore, and the results were prone to show a negative association between cholecystectomy and colon and rectal cancer risk compared with GSD patients without surgery. Thus, cholecystectomy may be recommended for patients with gallstones who meet the surgical indications. But the sample size incorporated in this study was relatively smaller; more cohort studies are encouraged in the future.

Besides, both GSD, with or without cholecystectomy, predispose to liver, pancreas, and biliary tract cancer risk compared with the health controls. The association may derive from the shared risk factors such as obesity [2] and a common mechanism of chronic inflammation [35]. Gallstones were additionally reported to cause mucosal damage and increase inflammatory mediators such as cytokine expression, which was further proposed as a carcinogenesis pathway [36, 37]. Additionally, results showed the risk for liver, pancreas, and biliary tract cancer trend to be lower among the cholecystectomy subgroup. Furthermore, results also suggested that when compared with GSD patients without surgery, cholecystectomy was not associated with pancreatic cancer and biliary tract cancer anymore. Therefore, timely treatment of GSD may protect patients from developing these cancers.

Contrary to the speculation that long‐time exposure to GSD may aggregate the cancer risk, meta‐regression indicates that with the follow‐up year increased, the strength of the associations between GSD and all causes of cancer incidence was decreased. Thus, the association between GSD and cancer may be partly overestimated with a relatively shorter follow‐up period. Subgroup analysis stratified by follow‐up cut‐offs indicates no reverse association, and lag‐time bias existed in the association between GSD and cancer risk, which further strengthened the causal associations. Additionally, the duration effect of GSD on gastrointestinal cancer could only last for 20 years, while the duration for liver, pancreas, and biliary tract cancer lasts for a lifetime; the different pathogenic mechanisms may explain these cancers caused by GSD, as was stated above. The results may shed light on the prevention strategies for the complications of GSD for clinical practice. After trim and fill adjustment, the associations between GSD and melanoma and lung cancer became significant. However, as shown in the TSA, this evidence was inconclusive and needed further verification.

### 4.3 Limitations

Several limitations are worth mentioning. Firstly, this study found substantial heterogeneity across studies; subgroups partly explained it, but it is not likely to be fully accounted for. It may somewhat affect the precision of the results, and therefore, the results should be explained with caution. Secondly, different gallstone subtypes (cholesterol, mixed, and pigmented) have disparate components derived from different pathological mechanisms. Thus, they may have a different effect on the longitudinal consequences. However, the subtypes of gallstones were not separately analyzed in the present study, as most of the studies could not provide detailed gallstone subtype information. Thirdly, although the present study included longitudinal studies that fully adjusted for the measurable confounding factors, it could not establish the causal associations because of the inherent weakness of the observational study design. In the future, we should conduct more randomized clinical studies or Mendelian randomization studies to confirm these causal associations. Finally, given that approximately 80% of individuals with GSD remain asymptomatic, the current diagnostic paradigm relying solely on medical records may introduce selection bias by over representing symptomatic cases. Consequently, future epidemiological investigations should incorporate population‐based screening methodologies to enhance the detection of asymptomatic gallstone carriers, thereby enabling a more precise assessment of the potential association between GSD and oncological risk.

## 5 Conclusion

The present meta‐analysis suggested that a history of GSD may increase the risk for all causes of cancer incidence, especially in hematologic malignancy, gastrointestinal cancer, liver, biliary, and pancreatic cancer, with sufficient evidence. It is essential to warrant clinicians and GSD patients to be aware of the subsequent outcomes. More screening strategies and prophylactic treatment approaches should be taken to prevent these complications. Furthermore, for GSD patients who meet the criteria, it was recommended to undergo cholecystectomy to prevent the patients from developing cancers. Therefore, it aims to improve survival rates and reduce the disease burden and economic cost.

## Author Contributions

**Wenqian Yu:** conceptualization (equal), writing – original draft (lead). **Jin Zhou:** writing – review and editing (equal). **Jing Xia:** conceptualization (equal), writing – original draft (supporting). **Jing Luo:** conceptualization (supporting), writing – original draft (supporting). **Shiyi Li:** conceptualization (supporting), writing – original draft (supporting). **Linjun Xie:** data curation (equal), visualization (equal). **YaZhou He:** data curation (equal), methodology (equal), visualization (equal). **Hongyu Li:** conceptualization (equal), writing – original draft (supporting). **Guoheng Jiang:** conceptualization (equal), writing – original draft (supporting). **Xin Chen:** software (equal). **Xuan Bai:** software (equal). **Min Mao:** validation (lead). **Xin Wang:** conceptualization (lead), writing – review and editing (lead).

## Ethics Statement

The study conducts a comprehensive analysis of previously published research results and does not involve direct human or animal research; hence, it does not require ethical approval.

## Conflicts of Interest

The authors declare no conflicts of interest.

## Supporting information


Appendix S1.

Appendix S2.

Appendix S3.

Appendix S4.

Appendix S5.

Appendix S6.

Appendix S7.

Appendix S8.

## Data Availability

The information included in the study is all contained in the article materials.
