# Supplementary material for: The Associations Between Gallstone Disease and Pan‐Cancer Incidence Risk Based on Over 13 Million Participants
Source: Cancer Med. 2025 Apr 25;14(9):e70857. doi: 10.1002/cam4.70857 (PMC12022677; doi:10.1002/cam4.70857)
Supplement: Supplementary file 1 — Appendix S1. [file CAM4-14-e70857-s003.docx]

# Appendix file-1: Literature search terms and equation.

(“gallstone” OR “gallstones” OR “gallstone disease” OR “cholelithiasis” OR “gallbladder stones” OR “gallbladder calculus” OR “biliary tract stones” OR “bile duct stones” OR “choledocholithiasis”)

# AND

(“odds ratio” OR “OR” OR “hazard risk” OR “hazard ratio” OR “HR” OR “relative risk” OR “RR” OR “rate ratio” OR “P” OR “P value” OR “P=” OR “association” OR “associated” OR “confidence interval” OR “CI” OR “censor” OR “Kaplan-Meier” OR “Cox model” OR “Proportional hazard model” OR “log-rank” OR “survival analysis”)

# AND

(“risk” OR “cohort” OR “nested case-control” OR “trial” OR “prospective” OR “follow-up” OR “registry” OR “record linkage” OR “longitudinal” OR “incidence”)
