# Supplementary material for: The Associations Between Gallstone Disease and Pan‐Cancer Incidence Risk Based on Over 13 Million Participants
Source: Cancer Med. 2025 Apr 25;14(9):e70857. doi: 10.1002/cam4.70857 (PMC12022677; doi:10.1002/cam4.70857)
Supplement: Supplementary file 2 — Appendix S2. [file CAM4-14-e70857-s004.docx]

Appendix file-2 List of included studies

A1. Johansen C, Chow WH, Jorgensen T, Mellemkjaer L, Olsen JH. [Risk of colorectal cancer and other cancer diseases in patients with gallstones]. Ugeskrift for laeger. 1998;160(6):831-5.

A2. Fall K, Ye W, Nyren O. Risk for gastric cancer after cholecystectomy. Am J Gastroenterol. 2007;102(6):1180-4.

A3. Kang SH, Kim YH, Roh YH, Kim KW, Choi CJ, Kim MC, et al. Gallstone, cholecystectomy and risk of gastric cancer. Ann Hepatobiliary Pancreat Surg. 2017;21(3):131-7.

A4. Lee J, Choe S, Park JW, Jeong SY, Shin A. The Risk of Colorectal Cancer After Cholecystectomy or Appendectomy: A Population-based Cohort Study in Korea. Journal of preventive medicine and public health = Yebang Uihakhoe chi. 2018;51(6):281-8.

A5. Shabanzadeh DM, Sorensen LT, Jorgensen T. Association Between Screen-Detected Gallstone Disease and Cancer in a Cohort Study. Gastroenterology. 2017;152(8):1965-74.e1.

A6. Park SM, Kim HJ, Kang TU, Kang MJ, Ahn HS. Effects of gallstones or cholecystectomy on the pancreatic cancer risk and prognosis: A population-based cohort study. Pancreas. 2019;48(10):1505.

A7. Ishiguro S, Inoue M, Kurahashi N, Iwasaki M, Sasazuki S, Tsugane S. Risk factors of biliary tract cancer in a large-scale population-based cohort study in Japan (JPHC study); with special focus on cholelithiasis, body mass index, and their effect modification. Cancer causes & control : CCC. 2008;19(1):33-41.

A8. Chen YK, Yeh JH, Lin CL, Peng CL, Sung FC, Hwang IM, et al. Cancer risk in patients with cholelithiasis and after cholecystectomy: a nationwide cohort study. Journal of gastroenterology. 2014;49(5):923-31.

A9. A M, JA M, LJ M, VS H, AR Z, EP D. Gallstones, gallbladder cancer, and other gastrointestinal malignancies. An epidemiologic study in Rochester, Minnesota. Annals of internal medicine. 1987;107(1):30-5.

A10. Nogueira L, Cross A, Freedman N, Lai G, Castro F, Koshiol J. Abstract 4804: Gallstones, cholecystectomy, and risk of digestive system cancers. 2013;73(8):-.

A11. Gudmundsson S, Moller TR, Olsson H. Cancer incidence after cholecystectomy--a cohort study with 30 years follow-up. European journal of surgical oncology : the journal of the European Society of Surgical Oncology and the British Association of Surgical Oncology. 1989;15(2):113-7.

A12. A E, CC H, J Y, D T, JK M, SJ L, et al. Risk of extrahepatic bileduct cancer after cholecystectomy. Lancet (London, England). 1993;342(8882):1262-5.

A13. A E, J Y, BM K, JK M, HO A. Risk of pancreatic and periampullar cancer following cholecystectomy: a population-based cohort study. Digestive diseases and sciences. 1996;41(2):387-91.

A14. A E, J Y, HO A, JK M, WH C, I P, et al. Cholecystectomy and colorectal cancer. Gastroenterology. 1993;105(1):142-7.

A15. A R, D M. Cholecystectomy and colon cancer in the elderly. Age and ageing. 1983;12(1):44-9. A16. A S, TM M, A P-H, RK R, BE H. A prospective study of pancreatic cancer in the elderly. International journal of cancer. 1994;58(1):46-9.

A17. AH W, A P-H, RK R, BE H. Alcohol, physical activity and other risk factors for colorectal cancer: a prospective study. British journal of cancer. 1987;55(6):687-94.

A18. ES S, DS M, MF L, E G, GA C, CS F. Gallstones, cholecystectomy, and the risk for developing pancreatic cancer. British journal of cancer. 2002;86(7):1081-4.

A19. G T, M F, C B, L C, A F, R S, et al. Previous cholecystectomy, gastrectomy, and diabetes mellitus are

not crucial risk factors for pancreatic cancer in patients with chronic pancreatitis. Pancreas. 2001;23(4):364-7.

A20. Goldacre MJ, Abisgold JD, Seagroatt V, Yeates D. Cancer after cholecystectomy: record-linkage cohort study. British journal of cancer. 2005;92(7):1307-9.

A21. Goldacre MJ, Wotton CJ, Abisgold J, Yeates DG, Collins J. Association between cholecystectomy and intestinal cancer: a national record linkage study. Annals of surgery. 2012;256(6):1068-72.

A22. GP N, A T, H T, H S. Cholecystectomy and colorectal carcinoma: a total-population historical prospective study. The American journal of gastroenterology. 1991;86(10):1486-90.

A23. Hartz A, He T, Ross JJ. Risk factors for colon cancer in 150,912 postmenopausal women. Cancer causes & control : CCC. 2012;23(10):1599-605.

A24. J F, W Y, E N, J L. Association between cholecystectomy and adenocarcinoma of the esophagus. Gastroenterology. 2001;121(3):548-53.

A25. JS A, JR A, DC D. The absence of a relationship between cholecystectomy and the subsequent occurrence of cancer of the proximal colon. Diseases of the colon and rectum. 1983;26(3):141-4.

A26. Kao WY, Hwang CY, Su CW, Chang YT, Luo JC, Hou MC, et al. Risk of hepato-biliary cancer after cholecystectomy: a nationwide cohort study. Journal of gastrointestinal surgery : official journal of the Society for Surgery of the Alimentary Tract. 2013;17(2):345-51.

A27. Lagergren J, Mattsson F. Cholecystectomy as a risk factor for oesophageal adenocarcinoma. The British journal of surgery. 2011;98(8):1133-7.

A28. Lagergren J, Mattsson F. Cholecystectomy and risk of laryngeal and pharyngeal cancer. International journal of cancer. 2012;130(9):2211-4.

A29. Lagergren J, Mattsson F, El-Serag H, Nordenstedt H. Increased risk of hepatocellular carcinoma after cholecystectomy. British journal of cancer. 2011;105(1):154-6.

A30. Lagergren J, Ye W, Ekbom A. No increased risk of breast cancer after cholecystectomy. International journal of cancer. 2000;88(4):679-81.

A31. Lagergren J, Ye W, Ekbom A. Intestinal cancer after cholecystectomy: is bile involved in carcinogenesis? Gastroenterology. 2001;121(3):542-7.

A32. Lai HC, Chang SN, Lin CC, Chen CC, Chou JW, Peng CY, et al. Does diabetes mellitus with or without gallstones increase the risk of gallbladder cancer? Results from a population-based cohort study. Journal of gastroenterology. 2013;48(7):856-65.

A33. Lai HC, Tsai IJ, Chen PC, Muo CH, Chou JW, Peng CY, et al. Gallstones, a cholecystectomy, chronic pancreatitis, and the risk of subsequent pancreatic cancer in diabetic patients: a population-based cohort study. Journal of gastroenterology. 2013;48(6):721-7.

A34. Li Q, Kuriyama S, Kakizaki M, Yan H, Nagai M, Sugawara Y, et al. History of cholelithiasis and the risk of prostate cancer: the Ohsaki Cohort Study. International journal of cancer. 2011;128(1):185-91. A35. Linos DimitriosA OFWM, Beart Robert JR, W. Beard C Mary, Dockerty Malcolm B, Kurland Leonard T Cholecystectomy and carcinoma of the colon. The Lancet. 1981:379-81.

A36. Nordenstedt H, Mattsson F, El-Serag H, Lagergren J. Gallstones and cholecystectomy in relation to risk of intra- and extrahepatic cholangiocarcinoma. British journal of cancer. 2012;106(5):1011-5.

A37. Peng YC, Lin CL, Sung FC. The association between cholecystectomy and colorectal neoplasm in inflammatory bowel diseases: A population-based cohort study. PloS one. 2017;12(5):e0177745.

A38. RA G, PA vdB, P vtV, E D, F S, RJ H. Cholecystectomy and colorectal cancer: evidence from a cohort study on diet and cancer. International journal of cancer. 1993;53(5):735-9.

A39. RZ S-S, P P, PR T, J V, D A. A prospective study of medical conditions, anthropometry, physical

activity, and pancreatic cancer in male smokers (Finland). Cancer causes & control : CCC. 2002;13(5):417-26.

A40. S G, HO A, O M, O N, UB K. Cholecystectomy as a risk factor for gastric cancer. A cohort study. Digestive diseases and sciences. 1984;29(2):116-20.

A41. Schernhammer ES, Leitzmann MF, Michaud DS, Speizer FE, Giovannucci E, Colditz GA, et al. Cholecystectomy and the risk for developing colorectal cancer and distal colorectal adenomas. British journal of cancer. 2003;88(1):79-83.

A42. Schmidt M, Smastuen MC, Sondenaa K. Increased cancer incidence in some gallstone diseases, and equivocal effect of cholecystectomy: a long-term analysis of cancer and mortality. Scand J Gastroenterol. 2012;47(12):1467-74.

A43. Shao T, Yang YX. Cholecystectomy and the risk of colorectal cancer. The American journal of gastroenterology. 2005;100(8):1813-20.

A44. Vogtmann E, Shu XO, Li HL, Chow WH, Yang G, Ji BT, et al. Cholelithiasis and the risk of liver cancer: results from cohort studies of 134,546 Chinese men and women. Journal of epidemiology and community health. 2014;68(6):565-70.

A45. W Y, J L, O N, A E. Risk of pancreatic cancer after cholecystectomy: a cohort study in Sweden. Gut. 2001;49(5):678-81.

A46. Ward HA, Murphy N, Weiderpass E, Leitzmann MF, Aglago E, Gunter MJ, et al. Gallstones and incident colorectal cancer in a large pan-European cohort study. International journal of cancer. 2019;145(6):1510-6.

A47. WH C, C J, G G, L M, JH O, JF F. Gallstones, cholecystectomy and risk of cancers of the liver, biliary tract and pancreas. British journal of cancer. 1999;79(null):640-4.

A48. Kiu KT, Chen HL, Huang MT, Sung CW, Liaw YP, Chang CC, et al. Outcome Analysis of Patients with Gallstone Disease Receiving Cholecystectomy: A Population-Based Cohort Study. Digestion. 2017;95(2):132-9.

A49. Chen CH, Lin CL, Kao CH. The Effect of Cholecystectomy on the Risk of Colorectal Cancer in Patients with Gallbladder Stones. Cancers (Basel). 2020;12(3).

A50. Chen CH, Lin CL, Kao CH. Association of Cholecystectomy with the Risk of Prostate Cancer in Patients with Gallstones. Cancers (Basel). 2020;12(3).

A51. Joshi AD, Nguyen LH, Gala M, Wirth J, Wu K, Giovannucci E, et al. Risk of colorectal cancer after cholecystectomy. Gastroenterology. 2018;154(6):S-334.
