# Supplementary figures and images for: The Associations Between Gallstone Disease and Pan‐Cancer Incidence Risk Based on Over 13 Million Participants
Source: Cancer Med. 2025 Apr 25;14(9):e70857. doi: 10.1002/cam4.70857 (PMC12022677; doi:10.1002/cam4.70857)

# Sensitivity Meta Analysis for All-cancer Incidence

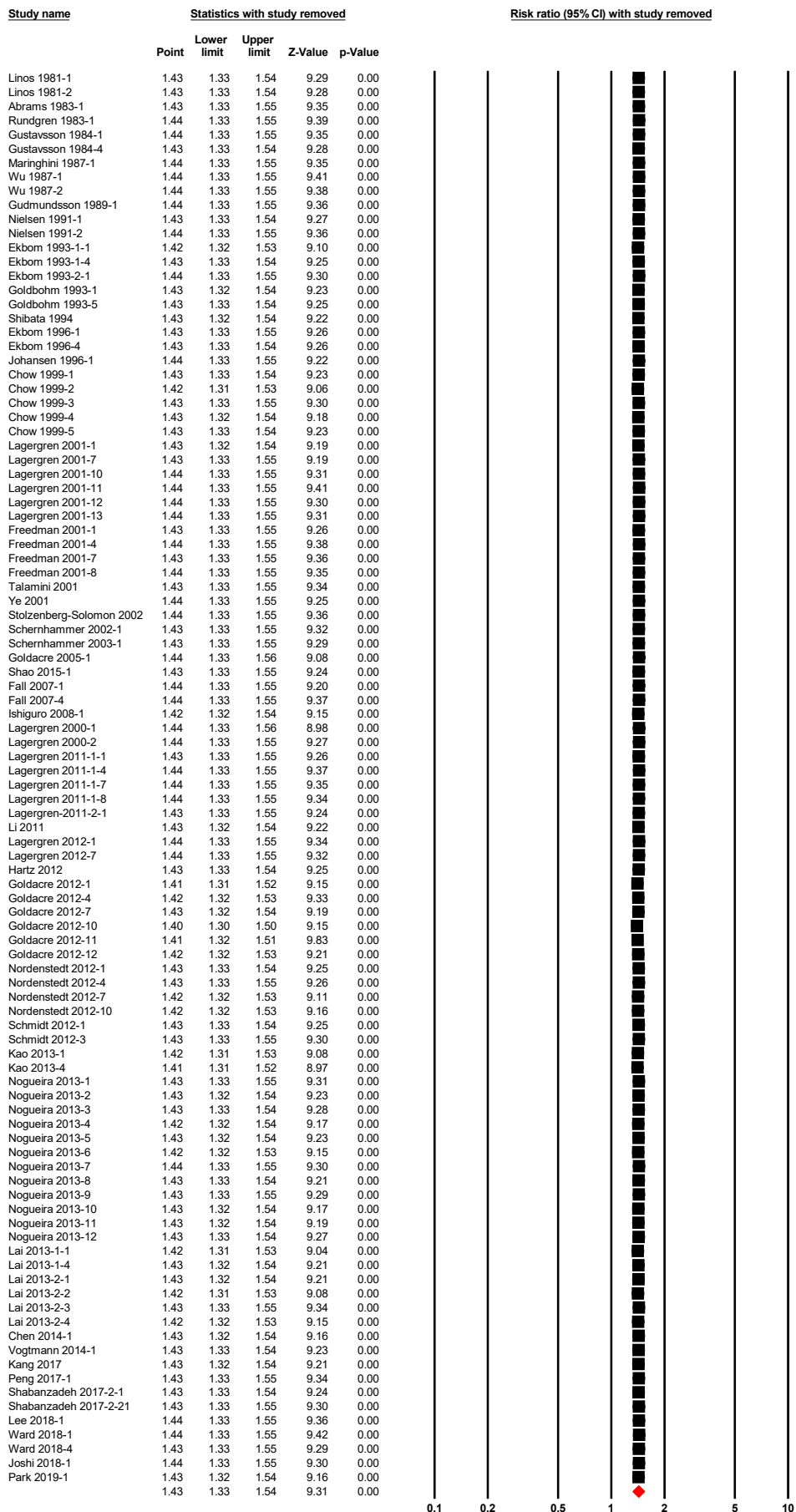

Supplement: Supplementary file 4 — Appendix S4. [file CAM4-14-e70857-s002.pdf]
