# Supplementary material for: The Associations Between Gallstone Disease and Pan‐Cancer Incidence Risk Based on Over 13 Million Participants
Source: Cancer Med. 2025 Apr 25;14(9):e70857. doi: 10.1002/cam4.70857 (PMC12022677; doi:10.1002/cam4.70857)

Appendix file-5: Subgroup analysis for the association between gallstone disease and non-communicable diseases and mortality stratified by sex.

Type of groups	No. of studies	Sample size	pooled RR (95% CI)	I^2 (%)	*p* for interaction
**Cancer incidence**					
Male	33	1856037	1.39(1.23−1.57)	92.91	0.18
Female	35	3668623	1.26(1.18−1.36)	91.17	
**Eye and nervous system cancer**					
Male	1	70	0.62(0.02−18.34)	0.00	0.88
Female	1	398	0.45(0.04−4.78)	0.00	
**Lip, oral cavity and pharynx cancer**					
Male	2	114968	1.06(0.87−1.28)	0.00	0.97
Female	2	230751	1.05(0.82−1.35)	0.00	
**Head and neck cancer**					
Male	1	70	0.89(0.02−37.15)	0.00	0.89
Female	1	398	1.23(0.07−23.04)	0.00	
**Hematologic malignancy**					
Male	1	70	0.55(0.05−5.83)	0.00	0.60
Female	1	398	1.13(0.32−4.04)	0.00	
**Lymphoma and myeloma cancer**					
Male	1	70	0.48(0.02−12.96)	0.00	0.65
Female	1	398	1.10(0.22−5.66)	0.00	
**Leukaemia**					
Male	1	70	0.65(0.02−18.94)	0.00	0.77
Female	1	398	1.18(0.15−9.13)	0.00	
**Skin cancer**					
Male	1	70	0.43(0.02−11.20)	0.00	0.94
Female	1	398	0.37(0.04−3.64)	0.00	
**Gastrointestinal Cancer**					
Male	19	927706	1.24(1.05−1.47)	93.52	0.91
Female	21	1981527	1.22(1.09−1.37)	90.20	
**Esophageal cancer**					
Male	4	203074	1.03(0.82−1.28)	95.03	0.98
Female	4	413540	1.05(0.92−1.20)	91.37	
**Gastric cancer**					
Male	4	88850	1.17(1.08−1.28)	95.51	0.78
Female	4	182646	1.00(0.92−1.09)	92.70	
**Small bowel cancer**					
Male	4	177040	3.07(1.19−7.92)	90.07	0.98
Female	4	431931	2.45(1.05−5.67)	76.87	
**Colorectal cancer**					
Male	15	637538	1.19(0.95−1.47)	94.79	0.99
Female	17	1389219	1.18(1.04−1.34)	91.06	
**Colon cancer**					
Male	12	625366	1.29(1.00−1.68)	95.15	0.80
Female	14	1376956	1.24(1.07−1.45)	92.19	

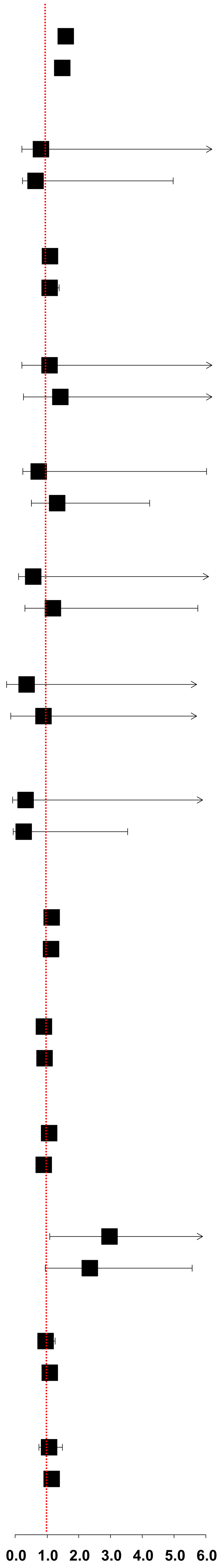

Type of groups	No. of studies	Sample size	pooled RR (95% CI)	I^2 (%)	*p* for interaction
**Rectal cancer**					
Male	9	533095	1.22(0.85−1.76)	88.66	0.83
Female	10	1036314	1.16(0.95−1.43)	74.07	
**Liver, pancreas and biliary tract cancer**					
Male	11	699745	1.95(1.55−2.45)	83.48	0.76
Female	12	1154632	1.86(1.49−2.31)	84.03	
**Liver cancer**					
Male	5	198347	1.60(1.05−2.45)	85.37	0.74
Female	5	349357	1.78(1.13−2.82)	83.89	
**Pancreatic cancer**					
Male	4	99564	1.22(1.02−1.45)	16.19	0.61
Female	3	148466	1.15(0.98−1.34)	0.00	
**Biliary tract cancer**					
Male	7	495357	2.95(2.12−4.12)	80.25	0.38
Female	7	695023	2.37(1.66−3.39)	87.46	
**Gallbladder cancer**					
Male	2	213683	5.04(2.91−8.72)	13.28	0.31
Female	2	209579	3.36(1.93−5.86)	3.08	
**Intrahepatic cholangiocarcinoma**					
Male	1	190014	2.42(1.50−3.90)	76.49	0.44
Female	1	348197	1.64(0.70−3.84)	93.35	
**Extrahepatic cholangiocarcinoma**					
Male	3	259481	2.24(1.26−4.00)	88.11	0.96
Female	3	443332	2.19(1.26−3.80)	91.92	
**Ampulla of Vater cancer**					
Male	1	20745	1.63(0.44−4.18)	0.00	0.89
Female	1	41870	1.46(0.59−3.01)	0.00	
**Urinary cancer**					
Male	1	70	1.06(0.06−17.43)	0.00	0.85
Female	1	398	1.46(0.25−8.53)	0.00	
**Respiratory cancer**					
Male	2	114968	0.97(0.82−1.14)	0.00	0.62
Female	2	230751	1.06(0.78−1.46)	0.00	
**Laryngeal cancer**					
Male	1	114898	0.97(0.82−1.14)	0.00	0.59
Female	1	230353	1.07(0.77−1.46)	0.00	
**Bone, connective and muscle cancer**					
Male	1	70	0.38(0.01−24.87)	0.00	0.89
Female	1	398	0.25(0.01−13.65)	0.00	

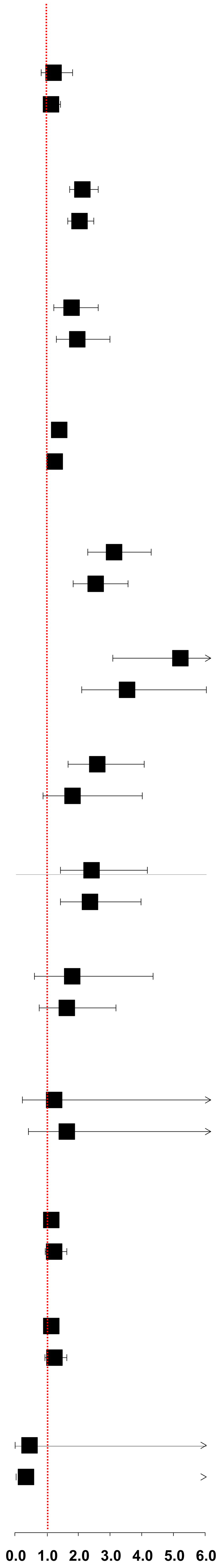

Supplement: Supplementary file 5 — Appendix S5. [file CAM4-14-e70857-s006.pdf]
