# Supplementary material for: The Associations Between Gallstone Disease and Pan‐Cancer Incidence Risk Based on Over 13 Million Participants
Source: Cancer Med. 2025 Apr 25;14(9):e70857. doi: 10.1002/cam4.70857 (PMC12022677; doi:10.1002/cam4.70857)

Appendix file-6 : Subgroup analysis for the associations between gallstone disease and non- communicable diseases and mortality stratified by geographic background.

Type of group	No. of studies	Sample size	pooled RR (95% CI)	I^2(%)	*p* for interaction
**Cancer incidence**					
America	10	1011666	1.33(1.19−1.49)	70.37	<0.01
Europe	27	5246186	1.34(1.22−1.48)	97.66	
Asia	11	6871426	2.16(1.77−2.64)	89.51	
**Eye and nervous system cancer**					
America	1	77725	1.88(0.76−4.63)	0.00	0.27
Europe	3	416633	1.12(0.93−1.35)	0.00	
**Brain cancer**					
America	1	416165	1.88(0.76−4.63)	0.00	0.27
Europe	2	77725	1.13(0.93−1.36)	0.00	
**Lip, oral cavity and pharynx cancer (Europe)**	3	387817	1.12(0.99−1.26)	10.19	NA
**Head and neck cancer**					
Europe	1	468	1.15(0.07−19.58)	0.00	0.98
Asia	1	77725	1.20(0.66−2.18)	67.31	
**Hematologic malignancy**					
Europe	3	416633	1.14(1.04−1.25)	0.00	0.98
Asia	1	77725	1.15(0.78−1.70)	0.00	
**Lymphoma and myeloma cancer (Europe)**	3	416633	1.08(0.96−1.22)	0.00	NA
**Leukaemia (Europe)**	3	416633	1.23(1.07−1.42)	0.00	NA
**Skin cancer**					
Europe	3	416633	1.03(0.99−1.07)	51.94	0.74
Asia	1	77725	1.14(0.64−2.02)	0.00	
**Melanoma**					
Europe	2	416165	0.78(0.61−0.99)	0.00	0.19
Asia	1	77725	1.83(0.52−6.45)	0.00	
**Gastrointestinal Cancer**					
America	8	843906	1.25(1.11−1.41)	65.87	0.90
Europe	17	3447821	1.28(1.13−1.46)	97.41	
Asia	4	125943	1.33(1.06−1.67)	52.74	
**Esophageal cancer**					
America	2	464333	1.29(0.96−1.73)	38.76	0.05
Europe	7	1413503	1.08(1.01−1.16)	41.82	
Asia	1	77725	0.92(0.50−1.70)	0.00	
**Gastric cancer**					
America	2	464333	1.47(1.11−1.95)	59.58	0.57
Europe	6	691002	1.75(1.37−2.23)	0.00	
Asia	2	124893	1.09(1.03−1.15)	0.00	
**Small bowel cancer**					
America	2	464333	1.94(1.43−2.65)	28.59	0.31
Europe	4	781600	3.51(1.85−6.65)	95.60	
Asia	1	77725	3.18(0.58−17.42)	0.00	
**Colorectal cancer**					
America	8	843906	1.15(1.02−1.30)	68.46	0.76
Europe	13	2188418	1.24(1.05−1.46)	97.80	
Asia	3	786438	1.21(1.03−1.42)	52.76	
**Colon cancer**					
America	6	832089	1.20(1.05−1.37)	71.73	0.69
Europe	13	2188430	1.30(1.08−1.57)	97.26	
Asia	2	708470	0.90(0.62−1.29)	28.49	
**Rectal cancer**					
America	3	216844	1.34(0.98−1.85)	36.09	0.54
Europe	11	708470	1.12(0.90−1.39)	95.21	
Asia	2	2186223	1.03(0.69−1.50)	0.00	
**Liver, pancreas and biliary tract cancer**					
America	4	632093	1.49(1.19−1.86)	57.05	<0.01
Europe	11	1747663	1.48(1.26−1.74)	92.22	
Asia	7	6800750	3.24(2.36−4.46)	92.22	
**Liver cancer**					
America	2	464333	1.68(1.32−2.14)	22.07	0.05
Europe	4	842228	1.28(1.18−1.39)	51.00	
Asia	3	214861	2.11(1.39−3.20)	90.21	

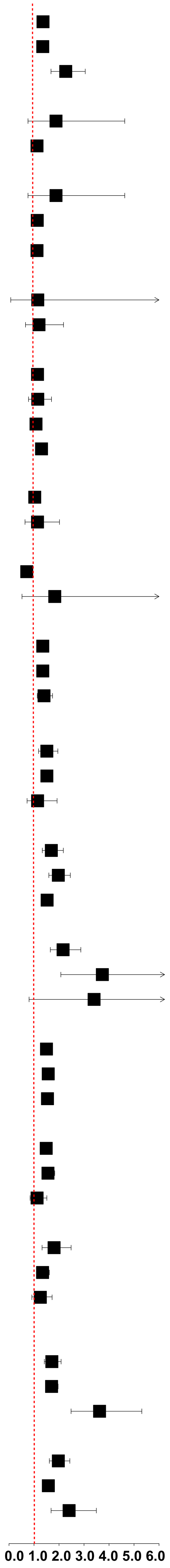

Type of group	No. of studies	Sample size	pooled RR (95% CI)	I^2(%)	*p* for interaction
**Pancreatic cancer**					
America	4	632093	1.41(1.05−1.89)	67.00	<0.01
Europe	7	800999	1.15(1.03−1.29)	54.36	
Asia	3	6140707	2.57(1.60−4.12)	87.45	
**Biliary tract cancer**					
America	1	2583	2.80(0.95−8.30)	0.00	0.04
Europe	4	723736	2.02(1.50−2.72)	92.74	
Asia	4	603222	5.70(2.70−12.01)	88.69	
**Gallbladder cancer**					
America	1	2583	2.80(0.94−8.35)	0.00	0.71
Europe	1	60176	3.60(2.62−4.94)	0.00	
Asia	3	600632	4.72(2.25−9.90)	68.45	
**Intrahepatic cholangio carcinoma (Europe)**	1	538211	1.95(0.99−3.86)	94.70	NA
**Extrahepatic bile duct cancer**					
Europe	3	661121	1.86(1.15−3.01)	95.94	0.28
Asia	2	179593	4.64(0.95−22.64)	87.60	
**Ampulla of Vater cancer(Europe)**	2	122791	1.89(1.29−2.77)	0.00	NA
**Urinary cancer**					
Europe	3	41663	1.05(0.97−1.14)	0.36	0.66
Asia	1	77725	0.97(0.70−1.35)	57.18	
**Kidney cancer**					
Europe	2	416165	1.18(1.02−1.37)	0.00	0.87
Asia	1	77725	1.23(0.79−1.91)	0.00	
**Bladder cancer**					
Europe	2	416165	1.00(0.91−1.10)	0.00	0.61
Asia	1	77725	0.74(0.46−1.20)	0.00	
**Respiratory cancer**					
Europe	4	761884	0.96(0.91−1.03)	0.00	0.73
Asia	1	77725	1.01(0.80−1.27)	0.00	
**Lung cancer**					
Europe	2	416165	0.96(0.89−1.03)	31.49	0.23
Asia	1	77725	1.00(0.79−1.27)	0.00	
**Laryngeal cancer**					
Europe	2	416165	0.98(0.86−1.13)	0.00	0.80
Asia	1	77725	1.13(0.40−3.19)	0.00	
**Bone, connective and muscle cancer (Europe)**	1	468	0.43(0.02−12.00)	0.00	NA
**Male specific cancer**					
Europe	4	388058	1.04(0.94−1.14)	0.00	0.02
Asia	2	57598	1.43(1.11−1.84)	5.72	
**Prostate cancer**					
Europe	4	201025	1.04(0.94−1.14)	0.00	0.02
Asia	2	57598	1.43(1.11−1.84)	5.72	
**Testis and penis cancer (Europe)**	2	10974	0.66(0.13−3.48)	0.00	NA
**Female specific cancer**					
Europe	5	504198	1.01(0.99−1.03)	49.92	0.44
Asia	1	42585	0.92(0.73−1.15)	0.00	
**Breast cancer**					
Europe	5	504198	1.01(0.99−1.03)	62.27	0.22
Asia	1	42585	0.92(0.73−1.15)	0.00	
**Cervical cancer**					
Europe	3	218626	0.99(0.71−1.40)	65.41	0.70
Asia	1	42585	1.11(0.70−1.76)	0.00	
**Ovary cancer (Europe)**	3	218626	0.93(0.79−1.10)	0.00	NA
**Uterine corpus cancer**					
Europe	3	218626	1.11(0.97−1.28)	0.00	0.75
Asia	1	42585	1.00(0.53−1.88)	0.00	
**Vagina, vulva cancer (Europe)**	1	398	2.56(0.19−34.96)	0.00	NA

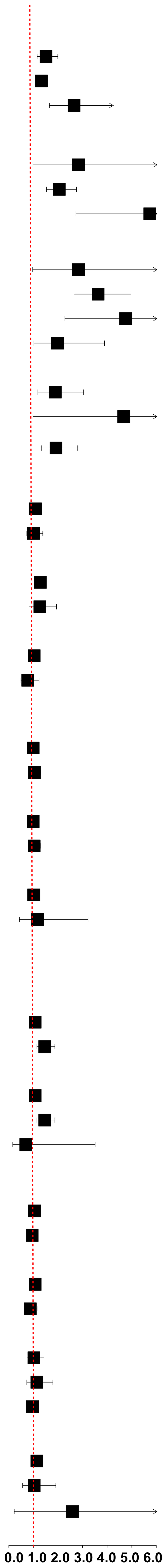

Supplement: Supplementary file 6 — Appendix S6. [file CAM4-14-e70857-s001.pdf]
