# Supplementary material for: The Associations Between Gallstone Disease and Pan‐Cancer Incidence Risk Based on Over 13 Million Participants
Source: Cancer Med. 2025 Apr 25;14(9):e70857. doi: 10.1002/cam4.70857 (PMC12022677; doi:10.1002/cam4.70857)

Appendix file-7: Subgroup analysis for the associations between gallstone disease and non- communicable diseases and mortality stratified by di fferent follow-up period.

Type of groups	No.of studies	Sample size	pooled RR (95% CI)	I^2 (%)	*p* for interaction
**Cancer incidence**					
3.1−5.0 years	5	941596	1.11(0.91−1.35)	75.43	<0.001
5.1−10.0 years	14	2474178	2.08(1.71−2.53)	98.48	
10.1−20.0 years	23	3781818	1.10(1.05−1.16)	84.49	
>20.0 years	6	262948	1.35(1.11−1.64)	88.65	
**Eye and nervous system cancer**					
5.1−10.0 years	1	42098	1.17(0.90−1.40)	0.00	0.64
10.1−20.0 years	2	451792	1.13(0.86−1.48)	26.85	
>20.0 years	1	468	0.39(0.04−3.93)	0.00	
**Brain cancer**					
5.1−10.0 years	1	42098	1.17(0.90−1.40)	0.00	0.84
10.1−20.0 years	2	451792	1.13(0.86−1.48)	26.85	
**Lip,oral cavity and pharynx cancer**					
5.1−10.0 years	1	42098	1.25(1.00−1.60)	0.00	0.33
10.1−20.0 years	1	345251	1.06(0.91−1.23)	0.00	
>20.0 years	1	468	0.28(0.01−5.98)	0.00	
**Head and neck cancer**					
10.1−20.0 years	1	77725	1.08(0.80−1.46)	67.31	0.97
>20.0 years	1	468	1.15(0.07−20.29)	0.00	
**Hematologic malignancy**					
5.1−10.0 years	1	42098	1.24(1.08−1.43)	0.00	0.28
10.1−20.0 years	2	451792	1.08(0.96−1.21)	0.00	
>20.0 years	1	468	0.92(0.27−3.07)	0.00	
**Lymphoma and myeloma cancer**					
5.1−10.0 years	1	42098	1.15(0.95−1.39)	0.00	0.72
10.1−20.0 years	1	374067	1.04(0.89−1.22)	0.00	
>20.0 years	1	468	0.90(0.19−4.27)	0.00	
**Leukaemia**					
5.1−10.0 years	1	42098	1.35(1.10−1.60)	0.00	0.41
10.1−20.0 years	1	374067	1.12(0.91−1.37)	0.00	
>20.0 years	1	468	0.94(0.14−6.49)	0.00	
**Skin cancer**					
5.1−10.0 years	1	42098	1.03(0.99−1.07)	85.64	0.54
10.1−20.0 years	2	451792	1.01(0.88−1.16)	0.00	
>20.0 years	1	468	0.30(0.03−2.76)	0.00	
**Melanoma**					
5.1−10.0 years	1	42098	0.76(0.60−1.00)	0.00	0.15
10.1−20.0 years	2	451792	1.41(0.63−3.15)	0.00	
**Gastrointestinal cancer**					
3.1−5.0 years	4	813188	1.27(1.14−1.41)	50.04	<0.01
5.1−10.0 years	7	851019	1.63(1.30−2.05)	97.99	
10.1−20.0 years	13	2037901	1.09(1.03−1.15)	65.19	
>20.0 years	4	137619	1.05(0.95−1.16)	0.00	
**Esophageal cancer**					
3.1−5.0 years	1	167646	0.97(0.72−1.31)	0.00	0.72
5.1−10.0 years	2	235058	1.10(0.94−1.29)	21.29	
10.1−20.0 years	6	1084711	1.07(0.92−1.25)	61.55	
>20.0 years	2	6396	0.63(0.21−1.92)	0.00	
**Gastric cancer**					
5.1−10.0 years	1	42098	1.07(0.90−1.27)	0.00	0.74
10.1−20.0 years	5	722820	1.11(1.05−1.17)	49.73	
>20.0 years	2	6392	0.85(0.38−1.87)	0.00	
**Small bowel cancer**					
5.1−10.0 years	2	502672	5.49(4.83−6.22)	92.32	<0.01
10.1−20.0 years	3	358768	1.78(1.40−2.27)	0.00	
>20.0 years	1	468	1.16(0.04−32.82)	0.00	
**Colorectal cancer**					
3.1−5.0 years	3	645542	1.32(1.17−1.48)	52.64	0.04
5.1−10.0 years	5	658059	1.49(1.17−1.90)	97.93	
10.1−20.0 years	10	1863556	1.05(0.99−1.12)	61.85	
>20.0 years	4	137619	1.06(0.96−1.17)	0.00	
**Colon cancer**					
3.1−5.0 years	2	637320	1.53(1.34−1.76)	0.00	<0.01
5.1−10.0 years	5	658584	1.56(1.10−2.23)	98.07	
10.1−20.0 years	8	1785582	1.06(0.98−1.14)	52.66	
>20.0 years	4	137619	1.35(0.97−1.86)	0.00	
**Rectal cancer**					
3.1−5.0 years	2	633974	1.04(0.89−1.21)	23.77	0.36
5.1−10.0 years	4	506904	1.38(1.02−1.88)	91.71	
10.1−20.0 years	6	1782041	1.03(0.90−1.18)	50.59	
>20.0years	3	136366	.114(0.92−1.42)	0.00	
**Liver, pancreas and biliary tract cancer**					
5.1−10.0 years	7	1600701	2.49(1.95−3.19)	90.86	0.01
10.1−20.0 years	10	1698742	1.54(1.25−1.89)	91.36	
>20.0 years	4	131745	1.49(1.00−2.21)	90.27	
**Liver cancer**					
5.1−10.0 years	3	197312	1.90(1.13−3.18)	94.25	0.44
10.1−20.0 years	4	799626	1.27(0.89−1.80)	86.98	
>20.0 years	1	62734	1.38(1.12−1.68)	0.00	

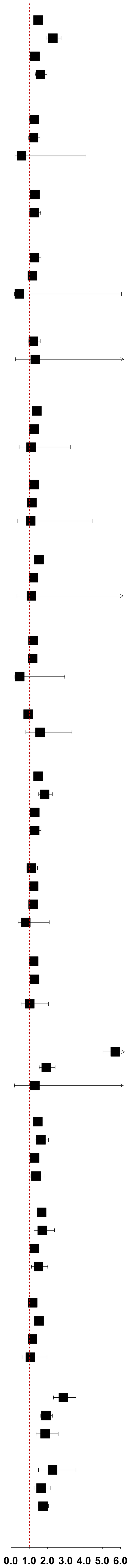

Type of groups	No.of studies	Sample size	pooled RR (95% CI)	I^2 (%)	*p* for interaction
**Pancreatic cancer**					
5.1−10.0 years	3	849566	2.34(1.41−3.86)	85.19	0.04
10.1−20.0 years	7	906372	1.21(0.94−1.54)	76.30	
>20.0 years	2	68543	1.20(1.06−1.36)	0.00	
**Biliary tract cancer**					
5.1−10.0 years	4	676765	2.99(2.18−4.11)	85.05	0.77
10.1−20.0 years	4	527427	2.85(1.45−5.62)	95.84	
>20.0 years	1	62734	2.30(1.21−4.37)	71.40	
**Gallbladder cancer**					
5.1−10.0 years	2	481215	3.82(3.00−4.95)	26.59	0.56
10.1−20.0 years	3	182176	5.74(1.55−21.30)	73.63	
**Intrahepatic cholangiocarcinoma**					
5.1−10.0 years	1	192960	2.77(2.17−3.49)	0.00	<0.01
10.1−20.0 years	1	345251	1.38(1.12−1.69)	0.00	
**Extrahepatic cholangiocarcinoma**					
5.1−10.0 years	2	253136	1.80(0.85−3.81)	91.66	0.45
10.1−20.0 years	3	524844	2.84(0.94−8.61)	91.60	
>20.0 years	1	62734	2.98(2.39−3.72)	0.00	
**Ampulla of Vater cancer**					
5.1−10.0 years	1	60176	2.10(1.30−3.10)	0.00	0.44
>20.0 years	1	62615	1.52(0.76−2.72)	0.00	
**Urinary cancer**					
5.1−10.0 years	1	42098	1.08(0.97−1.19)	56.94	0.71
10.1−20.0 years	2	451792	1.00(0.89−1.13)	10.66	
>20.0 years	1	468	1.33(0.30−5.91)	0.00	
**Kidney cancer**					
5.1−10.0 years	1	42098	1.21(1.01−1.45)	0.00	0.75
10.1−20.0 years	2	451792	1.16(0.92−1.45)	0.00	
**Bladder cancer**					
5.1−10.0 years	1	42098	1.02(0.90−1.15)	0.00	0.44
10.1−20.0 years	2	451792	0.95(0.82−1.09)	10.34	
**Respiratory cancer**					
5.1−10.0 years	1	42098	1.01(0.90−1.14)	0.00	0.67
10.1−20.0 years	3	797043	0.95(0.89−1.02)	0.00	
>20.0 years	1	468	0.96(0.24−3.80)	0.00	
**Lung cancer**					
5.1−10.0 years	1	42098	1.02(0.90−1.15)	0.00	0.26
10.1−20.0 years	2	451792	0.94(0.87−1.02)	0.00	
**Laryngeal cancer**					
5.1−10.0 years	1	42098	0.93(0.61−1.42)	0.00	0.78
10.1−20.0 years	2	422976	0.99(0.86−1.15)	0.00	
**Bone, connective and muscle cancer**					
>20.0 years	1	468	0.43(0.02−12.00)	0.00	NA
**Male specific cancer**					
5.1−10.0 years	2	33362	1.06(0.94−1.19)	63.07	0.23
10.1−20.0 years	3	222174	1.14(0.99−1.32)	0.00	
>20.0 years	2	3087	0.67(0.37−1.23)	0.00	
**Prostate cancer**					
5.1−10.0 years	2	33362	1.27(0.77−2.10)	80.73	0.23
10.1−20.0 years	2	222174	1.14(0.99−1.32)	0.00	
>20.0 years	2	3087	0.67(0.37−1.24)	0.00	
**Testis and penis cancer**					
5.1−10.0 years	1	10904	0.68(0.10−2.00)	0.00	0.94
>20.0 years	1	70	0.57(0.01−27.57)	0.00	
**Female specific cancer**					
3.1-5.0 years	1	128408	0.98(0.92−1.04)	0.00	0.14
5.1−10.0 years	1	31194	1.03(0.99−1.08)	57.03	
10.1−20.0 years	3	383902	1.00(0.98−1.03)	34.50	
>20.0 years	2	3279	1.41(1.05−1.89)	0.00	
**Breast cancer**					
3.1-5.0 years	1	128408	0.98(0.92−1.04)	0.00	0.02
5.1−10.0 years	1	31194	1.05(1.00−1.10)	0.00	
10.1−20.0 years	3	383902	0.94(0.84−1.06)	67.09	
>20.0 years	2	3279	1.49(1.08−2.05)	0.00	
**Cervical cancer**					
5.1−10.0 years	1	31194	0.84(0.70−1.10)	0.00	0.05
10.1−20.0 years	2	229619	1.21(0.96−1.52)	0.00	
>20.0 years	1	398	0.71(0.12−4.36)	0.00	
**Ovary cancer**					
5.1−10.0 years	1	31194	0.91(0.70−1.10)	0.00	0.97
10.1−20.0 years	1	187034	0.95(0.76−1.17)	0.00	
>20.0 years	1	398	0.94(0.24−3.66)	0.00	
**Uterine corpus cancer**					
5.1−10.0 years	1	31194	1.10(0.90−1.30)	0.00	0.95
10.1−20.0 years	2	229619	1.12(0.92−1.36)	0.00	
>20.0 years	1	398	0.90(0.23−3.44)	0.00	
**Vagina,vulva cancer (>20.0 years)**	1	398	2.56(0.19−34.96)	0.00	NA

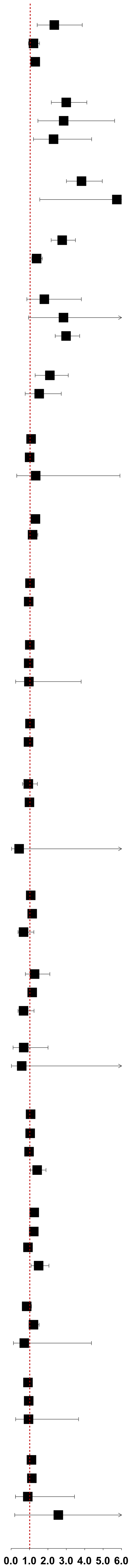

Supplement: Supplementary file 7 — Appendix S7. [file CAM4-14-e70857-s005.pdf]
