# Supplementary material for: The Associations Between Gallstone Disease and Pan‐Cancer Incidence Risk Based on Over 13 Million Participants
Source: Cancer Med. 2025 Apr 25;14(9):e70857. doi: 10.1002/cam4.70857 (PMC12022677; doi:10.1002/cam4.70857)

Appendix file-8 Trial sequence analysis for the eye and nervous system cancer(A), lip, oral cavity and pharynx cancer (B), headache and neck cancer (C), hematologic malignancy (D), skin cancer(E), gastrointestinal cancer (F), liver, biliary and pancreatic cancer(G), urinary cancer(H), respiratory cancer(I), male(J) and female (K) specific cancer.

A

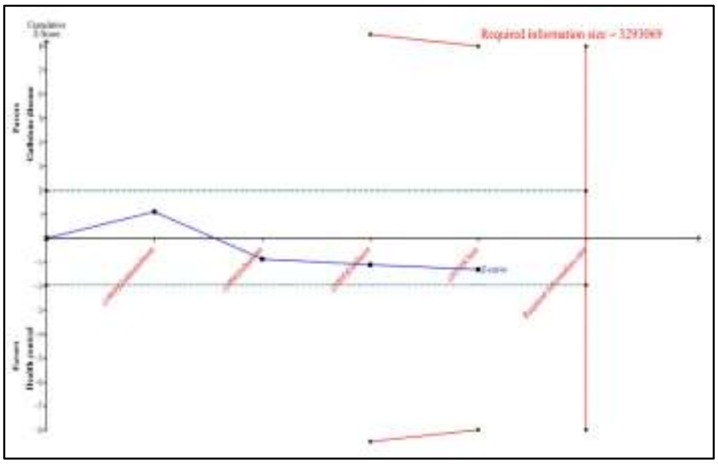

B

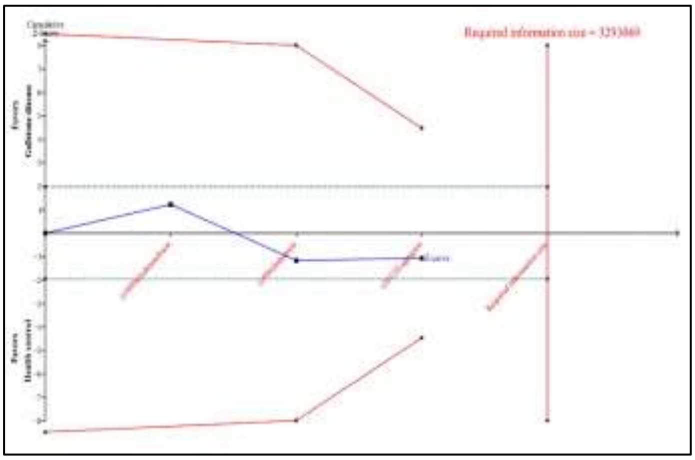

C

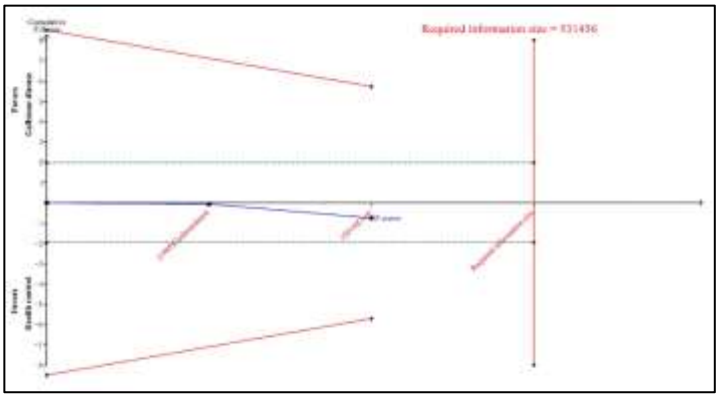

D

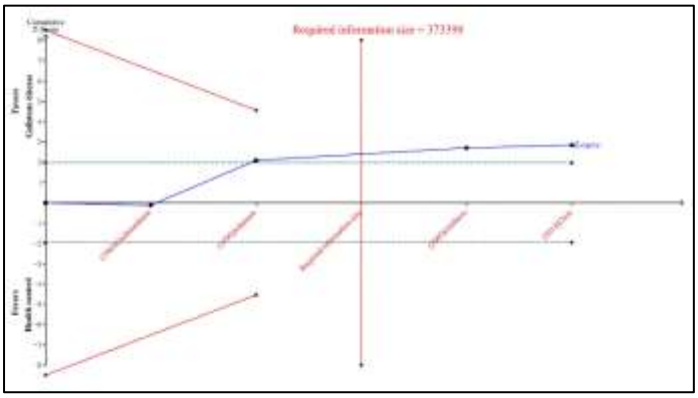

E

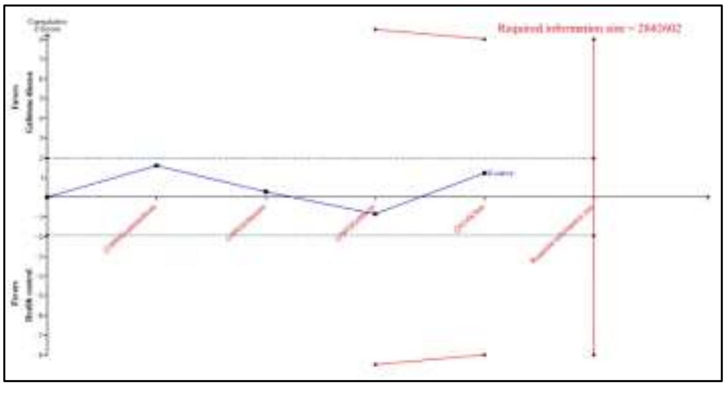

F

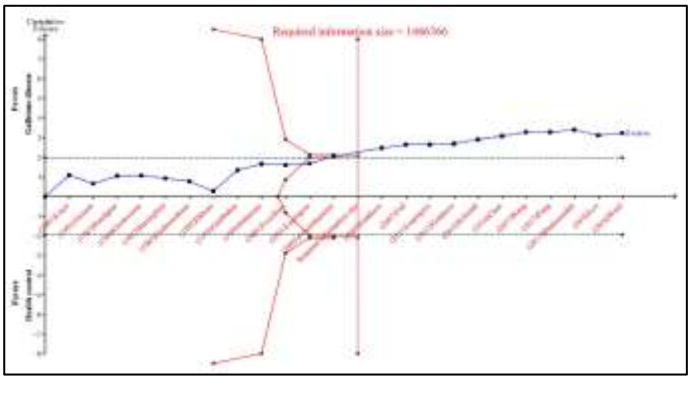

G

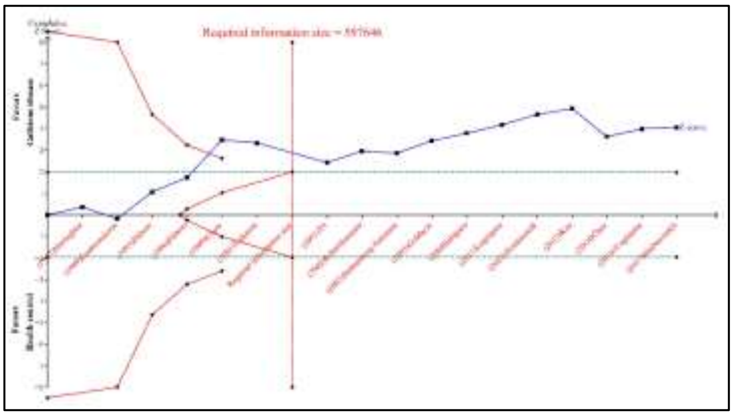

H

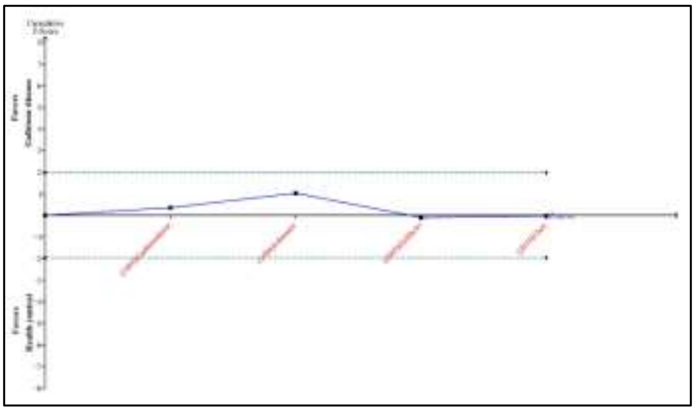

I

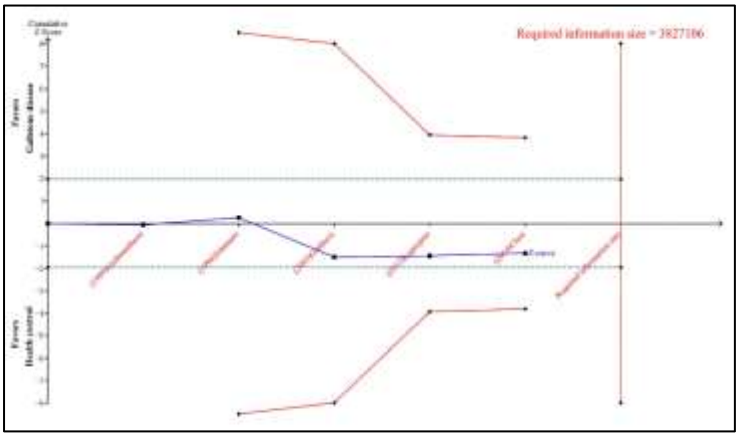
Required information size = 3827106

J

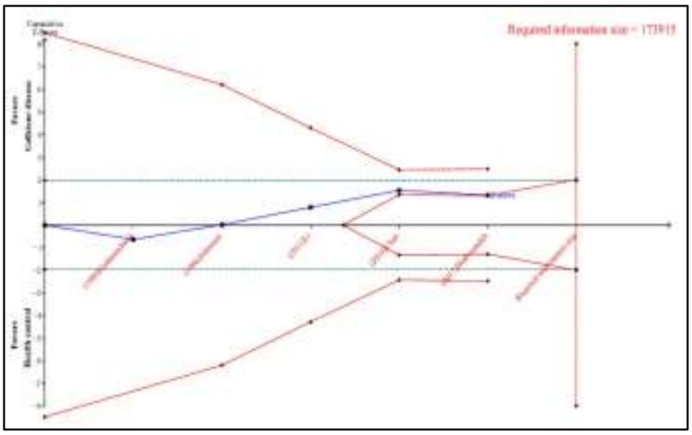
Required information size = 173915

K

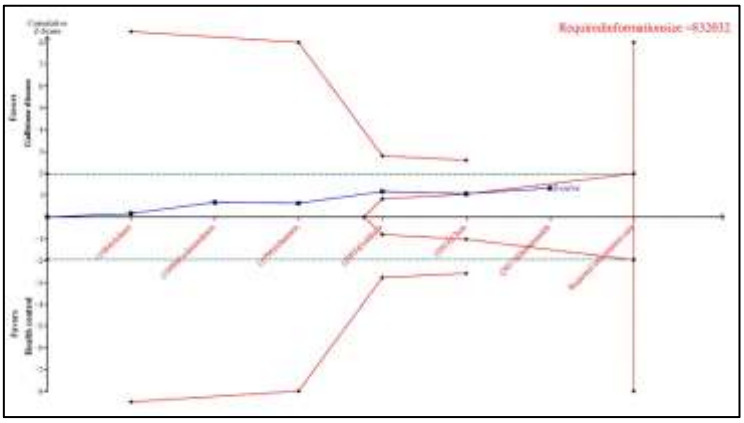

Supplement: Supplementary file 8 — Appendix S8. [file CAM4-14-e70857-s008.pdf]
